# Selective Oxidation of Hydrogen Sulfide to Sulfur Using Vanadium Oxide Supported on Porous Clay Heterostructures (PCHs) Formed by Pillars Silica, Silica-Zirconia or Silica-Titania

**DOI:** 10.3390/ma11091562

**Published:** 2018-08-30

**Authors:** Juan Antonio Cecilia, M. Dolores Soriano, Alejandro Natoli, Enrique Rodríguez-Castellón, José Manuel López Nieto

**Affiliations:** 1 Departamento Química Inorgánica, Cristalografía y Mineralogía, Facultad de Ciencias, Unidad Asociada al ICP-CSIC, Universidad de Málaga, 29071 Málaga, Spain; castellon@uma.es; 2 Instituto de Tecnología Química, UPV-CSIC, Campus Universidad Politécnica de Valencia, Avenida de los Naranjos s/n, 46022 Valencia, Spain; mdosorod@itq.upv.es (M.D.S.); jmlopez@itq.upv.es (J.M.L.N.); 3 Department of Materials and Ceramic Engineering, CICECO, University of Aveiro, 3810-193 Aveiro, Portugal; alejandro.natoli@gmail.com

**Keywords:** porous clay heterostructure, PCH, V_2_O_5_, H_2_S selective oxidation, sulfur elemental

## Abstract

Vanadium oxide (V_2_O_5_) species has been supported on different porous clay heterostructures (with silica pillars, silica-zirconia with a molar ratio Si/Zr = 5 and silica-titania with a molar ratio Si/Ti = 5) by wetness incipient method. All catalysts were characterized by X-ray diffraction (XRD), N_2_ adsorption-desorption at −196 °C, NH_3_ thermoprogrammed desorption (NH_3_-TPD), Raman spectroscopy, diffuse reflectance UV-Vis and X-ray photoelectron spectroscopy (XPS). After that, the catalytic activity of the vanadium-based catalysts was evaluated in the selective oxidation of H_2_S to elemental sulfur. The catalytic data show that both the activity and the catalytic stability increase with the vanadium content, obtaining the highest conversion values and sulfur yield for the catalysts with vanadium content of 16 wt.%. The comparison among all supports reveals that the incorporation of TiO_2_ species in the pillars of the PCH improves the resistance to the deactivation, attaining as best results a H_2_S conversion of 89% for SiTi-PCH-16V catalyst and elemental sulfur is the only compound detected by gas chromatography.

## 1. Introduction

The depletion of petroleum reserves has given rise to use of feedstock with lower quality and higher content of sulfur, nitrogen, oxygen and metals. The hydrotreating of crude oil fractions and the purification of natural gas produce large amounts of hydrogen sulfide (H_2_S), which is harmful to animals and humans [1] and extremely corrosive to piping and production facilities [2]. This fact has led to strong environmental regulations about H_2_S emissions into the atmosphere. Actually, the most common technology used is the Claus process [3], where H_2_S can be recovered in the form of elemental sulfur. However, this reaction displays thermodynamic limitations, due to the fact that between 3–5% of the H_2_S cannot be converted in elemental sulfur. In order to improve the efficient removal of H_2_S, several purification processes have been proposed, such as adsorption, absorption, wet oxidation, and selective catalytic oxidation [4]. Among these processes, selective catalytic oxidation of H_2_S to elemental sulfur using oxygen from air [3] has been the most attractive process to remove H_2_S of the tail gas because through this reaction, the thermodynamic limitations disappear. The oxidation capacity of the catalysts must be limited to avoid the reverse Claus reaction and the oxidation of elemental sulfur to undesired SO_2_.

Metal oxide-based catalysts (e.g., V_2_O_5_, Mn_2_O_3_, Fe_2_O_3_, CuO, CoO, Bi_2_O_3_, and TiO_2_) are the most tested in continuous process for the selective catalytic oxidation of H_2_S [5]. Among them, both iron and vanadium-based catalysts have received more attention in the last decade. Iron oxide-based catalysts are inexpensive materials which have shown high activity in H_2_S selective catalytic oxidation; however, the selectivity toward elemental sulfur is relatively low due to the excess oxygen requirements [6], although the use of a catalytic support leads to the formation of iron isolated species and small oligomers species, increasing the selectivity to elemental sulfur [7].

Vanadium oxides-based catalysts have been the most active phases reported in the literature for the H_2_S selective catalytic oxidation due to the high conversion and selectivity to elemental sulfur [8,9,10,11,12,13,14]. Vanadium oxide catalysts in the form of V_2_O_5_ nanocrystals suffer a partial reduction to form V_4_O_9_, by the treatment of V_2_O_5_ in H_2_S/air during the partial oxidation of H_2_S at ca. 200 °C. This process takes place in a very short time during the reaction, leading to V^5+^-O-V^4+^ pairs, which have been proposed as the active species in the selective oxidation of H_2_S. Nonetheless the catalytic activity decays with time on stream due to the depletion of the amount of labile oxygen species on the catalyst surface which are evolved in the redox behavior of the catalyst and the selective oxidation of the sulfur hydrogen [8].

The use of catalytic supports with high thermal and mechanical resistance together with a high surface area favors the dispersion of the active site leading to a higher number of available centers for the reaction. Nonetheless, the choice of an adequate support is a key parameter in the catalytic behavior. Therefore, SiO_2_, Al_2_O_3_, TiO_2_, ZrO_2_ or MgO have been proposed as support for the selective oxidation of H_2_S due to metal oxide support and the vanadium species strongly influence the catalytic behavior of the supported vanadium catalysts. Among them, SiO_2_ has shown a higher resistance to be sulfated easily by heating with H_2_S or SO_2_ with an excess oxygen [4]. The incorporation of heteroatoms such as Ti, Zr or Al into silica-based materials can modify the electronic density of the support. In this sense, it has been reported that selective oxidation of H_2_S takes place on acidic sites while the reverse Claus reaction proceeds on basic sites [4].

The need to develop new porous materials with high availability and low cost has led to the use of clay mineral as starting material to obtain porous material with high thermal and chemical resistance. The clay mineral is inexpensive, very abundant, non-toxic and very versatile material with a wide range of applications mainly as adsorbent and catalyst [15]; however, efficient use of the clay mineral is limited by its poor thermal stability and low porosity. The insertion of pillars between two adjacent layers of montmorillonite by the incorporation of polyoxocations leading to pillared interlayered clays (PILCs) provides an increasing of its porosity and thermal stability. In the case of the selective oxidation of H_2_S, Bineesh et al. have realized research in PILCs using polyoxocations of Fe [16], Zr [17,18], Al [19] and Ti [17,20,21] and vanadium oxide as active phase reaching higher conversion values at reaction temperatures lower than 300 °C, mainly for Ti polyoxocations where the highest conversion and selectivity toward elemental sulfur were observed.

Another synthetic strategy to obtain a porous material from montmorillonite was proposed by Galarneau et al. [22], which describes the formation of a porous clay heterostructure (PCH) by:

(1) a cationic exchange of Na^+^ by a bulky cation, which produces an expansion of the interlayer spacing
(2) the insertion of pillars by polymerization of alkoxides around the cationic micelle
(3) calcination of the organic template, obtaining porous materials with higher specific surface area and more resistant than PILCs [23]. From this synthetic process, it is possible to modulate the pore volume by the use of an adequate cation to expand the interlayer spacing. In addition, the incorporation of heteroatoms such as Zr, Ti or Al in the pillars improves the thermal stability and provides interesting properties of these materials as catalyst, catalytic support [10,24,25] and adsorbent [26,27].

The present research describes the synthesis and characterization of porous clay heterostructures with silica, silica-zirconia and silica-titania pillars. These materials were impregnated with vanadium oxide species and were evaluated in the selective oxidation of H_2_S to sulfur elemental. In the present work, we studied the influence of the acidity on the activity, selectivity and stability. In addition, the used catalysts were recovered to evaluate the changes in the oxidation state and subsequently the structure of the active phase during the reaction. 

## 2. Materials and Methods

### 2.1. Synthesis of the PCH

The starting material to synthesize the porous clay heterostructures was a raw bentonite obtained from Almería (Spain) and supplied by Minas de Gador S.A. (Almería, Spain). This material was previously characterized in several research papers [23,28] as displaying a large proportion of montmorillonite (>90 wt.%).

First, the starting bentonite was treated by a sedimentation process to purify the montmorillonite fraction. Thereafter, the montmorillonite was treated with a NaCl 1 M solution to obtain the homoionic montmorillonite (Na-mont).

PCHs were synthesized by the method described by Cecilia et al. [23] in which 10 g of homoionic montmorillonite was treated with a solution of 36 g of hexadecyltrimethylammonium bromide (HDTMABr) (Sigma-Aldrich, St. Louis, MO, USA) in 400 mL of *n*-propanol (99.9% VWR) under stirring to favor the cationic exchange (Na^+^ by HDTMA^+^), leading to the expansion of the interlayer spacing. After 3 days, the suspension was filtered and washed with water to remove the excess of HDTMA^+^ non-intercalated until a neutral pH was obtained in the wash water. The solid was recovered and suspended in 1000 mL of water and was stirred for 24 h. After this time a solution of 3.6 g of hexadecylamine (90 wt.%, VWR, Radnor, PA, USA), used as co-surfactant, dissolved in 100 mL of *n*-propanol, was added to the mother solution and stirred for 24 h. The pillars between adjacent sheets were generated by the incorporation to the mother solution of a solution of 0.2 mol of silicon, silicon/zirconium or silicon/titanium with a molar ratio Si/Ti or Si/Zr of 5 in the form of their respective alkoxides, which were previously dissolved in 40 mL of *n*-propanol. The obtained gel was stirred for 72 h and then was filtered and washed with water and ethanol and dried at 60 °C in air for 12 h. Finally, the surfactant was removed by the calcination at 550 °C with a rate of 1 °C min^−1^ during 6 h. The catalytic supports will be labeled as Si-PCH, for the PCH pillared with silica, SiZr-PCH, for the PCH pillared with silica-zirconia with a molar ratio Si/Zr = 5 and SiTi-PCH, for the PCH pillared with silica-titania with a molar ratio Si/Ti = 5.

In a next step, vanadium oxide species were incorporated to the Si-PCH, SiZr-PCH and SiTi-PCH using ammonium metavanadate (Sigma-Aldrich) as precursor salt. The incorporation of vanadium species was carried out by the wetness impregnation method and its subsequent drying at 60 °C overnight. These catalysts will be referred to as *x*-PCH-*y*V, where *x* represents the composition of the pillars of the PCH and *y* represents the percentage in weight of vanadium present in the sample. The samples were finally calcined at 400 °C for 2 h.

### 2.2. Catalysts Characterization

Powder patterns for the samples were collected on a X’Pert ProMPD automated diffractometer (PANalytical B.V., Almelo, The Netherlands) equipped with a Ge (1 1 1) primary monochromator (strictly monochromatic Cu Kα radiation) and an X’Celerator detector. The diffractograms were recorded in the range of 2θ of 1–70° with step size of 0.017°.

The textural parameters (S_BET_ and V_P_) were evaluated from the nitrogen adsorption-desorption isotherms at −196 °C by an automatic ASAP 2020 system from Micromeritics (Norcross, GA, USA). Prior to the measurements, samples were outgassed at 200 °C and 10^−4^ mbar overnight. Surface areas were determined using the Brunauer–Emmett–Teller (BET) equation and a nitrogen molecule cross section of 16.2 Å^2^. The total pore volume was calculated from the adsorption isotherm at P/P_0_ = 0.996. Pore-size distribution was determined using the density functional theory (DFT).

The temperature programmed desorption of ammonia (NH_3_-TPD) was carried out to evaluate the total acidity of the supports and the vanadium-based catalysts. Prior to the adsorption of NH_3_, 80 mg of each sample was heated from room temperature to 550 °C (heating rate: 10 °C min^−1^) and then maintained at 550 °C during 10 min under flowing He (35 mL min^−1^). Subsequently, samples were cooled to 100 °C and then were exposed to flowing pure ammonia for 5 min. With the aim to remove the physisorbed ammonia, samples were cleaned using He flow (35 mL min^−1^) again. NH_3_-TPD was performed between 100 and 550 °C with a heating rate of 10 °C min^−1^ using a He flow and maintained at 550 °C for 15 min. The desorbed ammonia was analyzed by online gas chromatography (Shimadzu GC-14A, Tokyo, Japan) provided with a thermal conductivity detector (TCD).

Raman spectra were recorded in ambient conditions using a (Renishaw system 1000, Wotton-under-Edge, UK) “in via” attached to a microscope. An argon ion laser (785 nm) was used as the excitation source and was typically operated at a power of 20 mW. Spectra were collected using a backscattering geometry with a 180° angle between the illuminating and the collected radiation.

UV-vis spectroscopy studies were carried out in the diffuse reflectance mode with a (Shimadzu MPC3100, Tokyo, Japan) spectrophotometer and BaSO_4_ as reference.

X-ray photoelectron spectra were collected using a Physical Electronics PHI 5700 spectrometer (Chanhassen, MN, USA) with non-monochromatic MgKα radiation (300 W, 15 kV, and 1253.6 eV) with a multi-channel detector. Spectra of pelletized samples were recorded in the constant pass energy mode at 29.35 eV, using a 720 μm diameter analysis area. Charge referencing was measured against adventitious carbon (C 1*s* at 284.8 eV). A PHI ACCESS ESCA-V6.0 F software package (version 6.0) was used for acquisition and data analysis. A Shirley-type background was subtracted from the signals. Recorded spectra were always fitted using Gaussian–Lorentzian curves in order to determine the binding energies of the different element core levels more accurately.

Temperature-programed reduction with H_2_ (H_2_-TPR) experiments were carried out using 0.08 g of freshly calcined catalyst placed in U-shaped quartz reactor inside a tubular oven. In order to remove any contaminant, the powders were pre-treated with helium (50 mL min^−1^) to 550 °C for 1 h. After cooling to ambient temperature, TPR experiments were carried out in 10 vol.% H_2_/Ar (35 mL min^−1^) increasing the temperature from room temperature to 800 °C with a heating ramp of 10 °C min^−1^, using a temperature programmable controller. The water produced in the reduction was removed with an isopropanol–liquid N_2_ trap. Hydrogen consumption was registered using a TCD, mounted in a Shimazdu 14-B gas chromatograph.

### 2.3. Catalytic Tests

Catalytic tests were carried out at atmospheric pressure in a fixed-bed quartz tubular flow reactor at 180 °C, controlled by a thermocouple. A catalyst weight of 0.05 g (40–60 mesh) was mixed with silicon carbide as inert to obtain an adequate bed height to quench the possible homogeneous reactions. A feed mixture composed of H_2_S/air/He with molar composition of 1.2/5.0/93.8, a total flow of 130 mL min^−1^ was passed through the reactor and a contact time (W/F) of 62.4 g_cat_ h^−1^ mol^−1^_H2S_ since these conditions were established as optima in previous research [8,9,10].

Analysis of reactants and products was carried out online by gas chromatography using two different chromatographic columns [9,10]: (i) Molecular Sieve 5A (for O_2_ and N_2_); and (ii) Porapak T (for H_2_S and SO_2_). The gas line after the reactor has been heated to 150 °C in order to avoid condensation of sulfur. Previous to venting the gases sulfur has been collected on a cold trap.

## 3. Characterization of the Catalysts

### 3.1. X-ray Diffraction

As was indicated in previous research, the XRD pattern of the raw bentonite (Supplementary Materials Figure S1) displays montmorillonite as the main crystallographic phase [23,28]. In addition, other phases such plagioclase, cristobalite, calcite and quartz are also detected.

**Figure 1:**
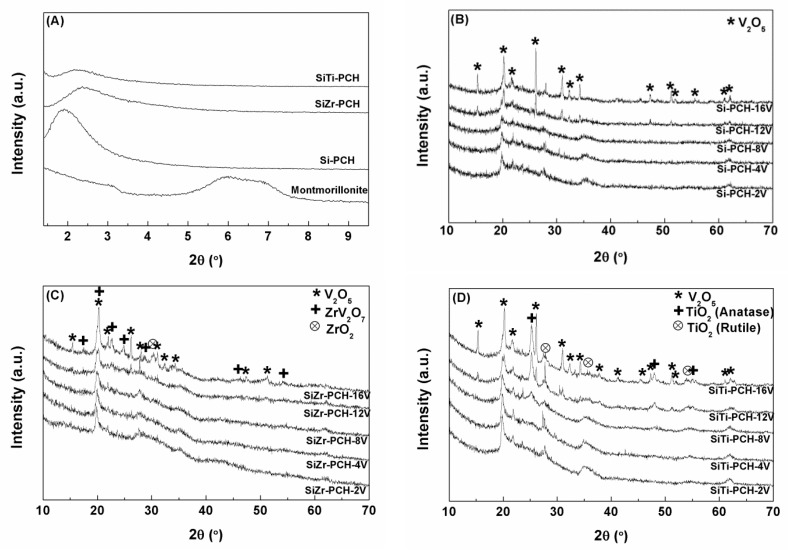
X-ray diffractograms of the supports (**A**), of the V-based catalysts supported on Si-PCH (**B**), of the V-based catalysts supported on SiZr-PCH (**C**) and of the V-based catalysts supported on SiTi-PCH (**D**).

After the purification process to obtain the montmorillonite phase and the formation of the PCHs, the XRD profiles of the Si-PCH, SiZr-PCH and SiTi-PCH supports differ from the raw bentonite one. The low-angle diffractograms of the supports (Figure 1A) reveal the presence of a broad band located between 2θ = 2–2.5° in all PCHs, which is assigned to the d_001_ reflection and is attributed to the expansion of the interlayer spacing. These layers are held by the pillars of silica, silica-zirconia or silica-titania in each case. The incorporation of small amounts of aluminum, zirconium or titanium species in the pillars causes a decrease of the intensity of the diffraction peak. In this sense, previous researches have reported that the addition heteroatoms favors the delamination of the montmorillonite leading to a more disordered structure [23,24,29].

The high-angle diffractograms of the catalytic supports (Supplementary Materials Figure S2) show how the basal reflections disappear while the non-reflections (020, 110, 200, 060) are noticeable after the PCHs formation confirming that the pillaring process takes place along the *c-*axis, as was expected. Nonetheless, the intensities of the non-basal reflections also decrease slightly due to the formation of the pillars causes a random displacement between layers along the *a-* and *b-*axis corroborating the formation of a more disordered structure like a house of cards [29].

XRD patterns of the vanadium oxide catalysts supported on Si-PCH (Figure 1B) display the presence of diffraction peaks located at 2θ = 15.4, 20.2, 21.7, 26.1, 31.1, 32.3, 34.3, 47.3, 51.2, 52.0, 55.6, 61.2 and 62.0°, which have been attributed to the presence of V_2_O_5_ species (PDF N: 01-076-1803) as unique crystallographic phase. From these diffractograms, one can observe how the intensity of the diffraction peaks of the V_2_O_5_ phase increases with the vanadium loading.

With regard to the vanadium oxide catalysts supported on SiZr-PCH (Figure 1C), the typical diffraction peaks, assigned to V_2_O_5_-species, are also detected. The intensity of these diffraction peaks increases with the vanadium content. Moreover, the diffractograms reveal the presence of diffraction peaks attributed to ZrV_2_O_7_ (PDF N: 98-008-4884) and ZrO_2_ (PDF N: 98-009-9744). In this sense, previous research has detected the extraction of zirconium species of Zr-MCM-41, which interacts with WO_x_ species leading to WO_x_/ZrO_2_ nanoparticles [30], so it is expected a similar extraction process of zirconium species from the pillars of the PCH which interacts with the vanadium species or remains as segregated ZrO_2_. In the same way, the vanadium oxide catalysts supported in SiTi-PCH (Figure 1D) also exhibit the diffraction peaks attributed to the V_2_O_5_ species and extracted titanium species from the SiTi-PCH support to generate TiO_2_ in the form of rutile (PDF N: 98-020-0391) and anatase (PDF N: 98-020-0392).

Finally, in all cases (Figure 1B–D), it is noticeable that the non-reflections attributed to montmorillonite layers of the PCH gradually disappear when the vanadium content increases. This fact suggests that higher vanadium content can affect to the porous framework of the catalytic support.

### 3.2. N_2_ Adsorption-Desorption at −196 °C

According to the IUPAC classification, refined by Rouquerol et al. [31], all PCHs exhibit a combination of type I and type IIb isotherms (Supplementary Materials Figure S3). These isotherms are associated to the presence of microporosity and the latter with monolayer-multilayer adsorption on an open and stable external surface of a powder with macroporosity, which are obtained in aggregates of plate-like particles that possess non-rigid slit-shaped pores. With regard to the hysteresis loop, all PCHs display a H3 type hysteresis loop with no indication of a plateau at high P/P_0_, which is attributed to agglomerates of particles forming slit-shaped pores (plates or edged particles like cubes) [24,32]. The incorporation of Zr or Ti into the pillars causes shrinkage of the hysteresis loop leading to a framework with blind cylindrical, cone-shaped and wedge-shaped pores [32], which is directly related with a house of cards framework typical of delaminated clay minerals [29].

The textural properties of the starting bentonite, catalytic supports and catalysts were evaluated by N_2_ adsorption-desorption at −196 °C (Table 1). Considering that the starting bentonite displays a S_BET_ of 49 m^2^ g^−1^, a considerable increasing of the specific surface area takes place after the cationic exchange by the substitution of Na^+^ cations by a bulky cation and the formation of pillared framework, obtaining S_BET_ values of 644, 608 and 562 m^2^ g^−1^ for Si-PCH, SiZr-PCH and SiTi-PCH, respectively. With regard to the pore-size distribution, Si-PCH support displays a higher proportion of micro- and mesoporosity than SiZr-PCH and SiTi-PCH ones. It has been reported in the literature that the incorporation of heteroatoms such as Zr or Ti favors the delamination of the sheets of montmorillonite [22,24], leading to a loss of the microporosity so a proportion of its S_BET_ is attributed to the interparticles porosity by the formation of a house of cards framework, mainly in the case of SiTi-PCH support [29] (Supplementary Materials Figure S4).

The addition of V_2_O_5_ species into the PCHs produces a progressive decrease of the S_BET_ and the pore volume in all supports (Table 1) due to the V_2_O_5_ crystals plug the meso- and mainly microporous structure of the PCHs, remaining the macroporosity attributed to the interparticle space (Supplementary Materials Figures S3 and S4). Despite the loss of the specific surface area, these catalysts display a higher dispersion of the active phase than the V_2_O_5_ bulk catalyst, which implies a higher amount of available active sites for the selective oxidation of the H_2_S.

### 3.3. NH_3_-Thermoprogrammed Desorption

The amount of acid sites of the starting montmorillonite, supports and catalysts was evaluated by NH_3_-TPD (Table 1). Clay minerals such as montmorillonite display itself acid sites due to the isomorphic substitution of aluminum by silicon, which generates a deficiency charge that leads to the formation of acid sites. In addition, it is well recognized in the literature that the incorporation of heteroatoms such as Al, Ti or Zr into de siliceous framework can also enhance the amount of acid sites of a porous material [23,33]. Thus, the Si-PCH displays an acidity of 308 µmol g^−1^, while the incorporation of titanium and zirconium provokes an increasing of the amount of available acid sites, being 395 µmol g^−1^ for the SiTi-PCH support and 460 µmol g^−1^ for the SiZr-PCH support.

The incorporation of a second heteroatom such as vanadium on the surface of the PCHs increases further the amount of acid sites [34]. Nonetheless, the loss of microporosity leads to the loss of the available acid sites and the decrease of the dispersion of the V_2_O_5_ species, although the acid density enhances, when the vanadium content increases.

### 3.4. Raman Spectroscopy

**Figure 2:**
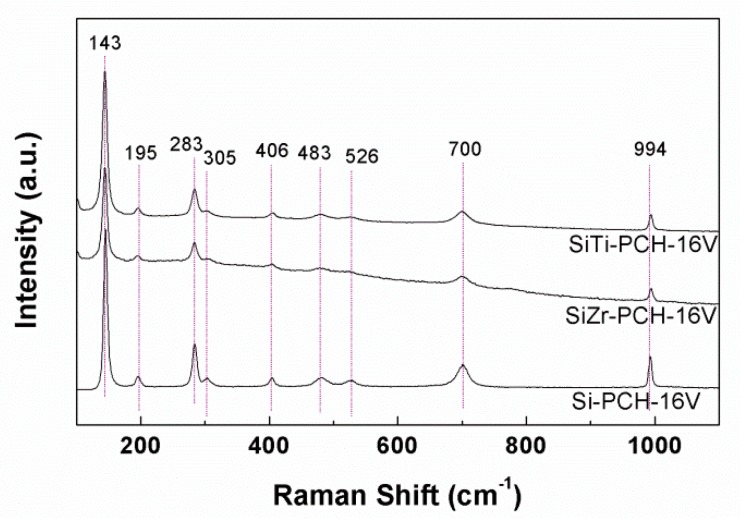
Raman spectra of Si-PCH-16V, SiZr-PCH-16V and SiTi-PCH16V catalysts.

For the Raman spectroscopy, it has been chosen *x*-PCH-16V catalysts as representative spectra (Figure 2) due to the catalysts with a V-loading lower than 8 wt.% do not show well-defined bands which suggests a high dispersion of the vanadium species on the surface of the PCHs support. All spectra display bands at 994, 700, 526, 483, 406, 305, 283, 195 and 143 cm^−1^, which are attributed to the vibrational modes of the V_2_O_5_ species [35]. Other crystalline species, shown in the XRD diffractograms of SiZr-PCH-yV and SiTi-PCH-yV catalysts (Figure 1C,D) such as ZrO_2_ or TiO_2_ have not been detected by Raman spectroscopy.

### 3.5. Diffuse Reflectance UV-Vis

**Figure 3:**
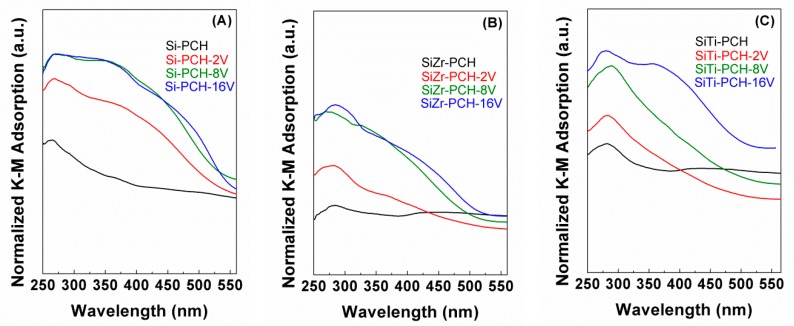
Diffuse reflectance UV-vis of the V-based catalysts supported on Si-PCH (**A**), of the V-based catalysts supported on SiZr-PCH (**B**) and of the V-based catalysts supported on SiTi-PCH (**C**).

Diffuse reflectance UV-vis spectra of the catalysts are compiled in Figure 3. All samples display spectra formed by several overlapped bands in the range of 250–575 nm, which are attributed to the presence of low energy O^2−^ to V^5+^ charge transfer. In this sense, previous research has established that the absorption band located between 250 and 290 nm is assigned to the presence of isolated tetrahedral V^5+^ species. The band located between 290 and 370 nm is attributed to the presence of associated vanadium species. Finally, the band above 470 nm is attributed to the presence of “*bulk-type*” polymeric species [36]. This band increases with the vanadium content for all supports indicating the formation of higher V_2_O_5_ clusters as was indicated in XRD data previously (Figure 1B–D). In addition, it is noticeable the absence of bands at higher energies (600–800 nm), which discards the d-d transition of V^4+^ species.

### 3.6. X-ray Photoelectronic Spectroscopy (XPS)

The chemical composition on the surface of the catalysts was evaluated by XPS (Table 2 and Table 3). The main band is observed in the O 1*s* region. This band can be deconvoluted in two contributions. The first one located about 532.5 eV has been assigned to the presence of oxygen species in the form of silica coming from the pillars and montmorillonite layers. The second contribution located about 530.5 eV is attributed to oxygen species in the form of vanadium oxide [10]. This contribution shows a lower intensity than the first one although increases its intensity when the vanadium content increases for all catalysts. The Si 2*p* region displays a single band located between 103.0–103.4 eV attributed to the silica species. In addition, the Al 2*p* core level spectrum displays a band located about 74.7 eV attributed to the presence of their respective oxide specie located in the octahedral and tetrahedral positions. In addition, it is noteworthy the absence of bands located in the Na 1*s* and Ca 2*p* regions, which confirms the total substitution of Na^+^ and Ca^2+^ by HDTMA^+^ in the synthesis step. The modified PCHs with titanium or zirconium in their pillars display an additional band in the Ti 2*p*_3/2_ region at 458.3 eV and Zr 3*d*_3/2_ region at 182.4 eV, which are attributed to the presence of Ti in the form of TiO_2_ and Zr in the form of ZrO_2_. After the impregnation and calcination of the vanadium species supported in PCHs arises a new band in the V 2*p*_3/2_ region with two contributions. The main contribution located about 517.1–517.5 eV has been ascribed the presence of V^5+^ species in the form of V_2_O_5_, while the other contribution located about 516.0–516.4 eV has been assigned to the presence of V^4+^ species which are formed by the thermal reduction or photoreduction of the V^5+^ species even though short exposition times were used, although this reduction diminishes with the vanadium content.

The atomic concentration data of the catalysts show how the vanadium content on the surface increases directly with the vanadium content mainly for SiZr-PCH and SiTi-PCH supports. In addition, the atomic concentration data also reveal that the concentration aluminum on the surface of the catalysts is higher in the case of SiTi-PCH and SiZr-PCH support in comparison with Si-PCH. Both facts can be in concordance with the higher delamination of SiTi-PCH and SiZr-PCH by the presence of zirconium and titanium in the pillars leading to an increasing of the meso- and macroporosity and a higher availability of the montmorillonite layers.

### 3.7. H_2_-Temperature Programmed Reduction

**Figure 4:**
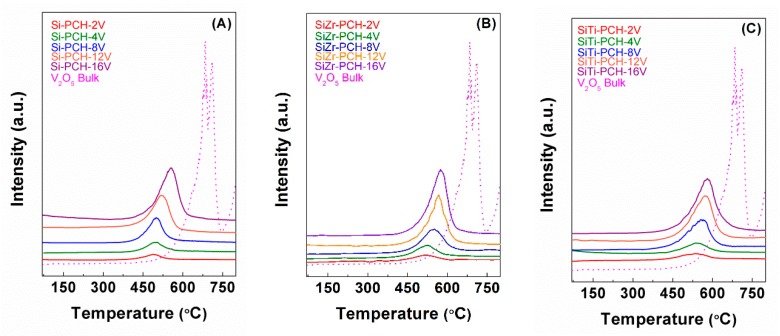
H_2_-TPR of the V-based catalysts supported on Si-PCH (**A**), of the V-based catalysts supported on SiZr-PCH (**B**) and of the V-based catalysts supported on SiTi-PCH (**C**).

The reducibility of the vanadium oxide-based catalysts has been tested by H_2_-TPR profiles (Figure 4). All H_2_-TPR profiles display a broad band, which has been attributed to the reduction of V^5+^ to V^3+^ species. This band is shifted to higher temperature when the vanadium content increases due to the loss of the dispersion and the formation of bigger crystals of vanadium species as was indicated in XRD (Figure 1B–D). The presence of titanium or zirconium in the pillared framework affects to the reducibility of the vanadium species, taking place a shift of the reduction of V^5+^ at higher temperature. This fact can be attributed to an increase of the vanadium particles as indicated the higher vanadium concentration on the surface when is used SiZr-PCH and SiTi-PCH as support (Table 2). In addition, the incorporation of heteroatoms such as zirconium and titanium generates acid sites (Table 1), which favors a higher interaction between the active phase and support. In this sense, Bineesh et al. have carried out H_2_-TPR for V/Ti-PILC obtaining reduction temperatures slightly lower to those shown in this work, confirming the high interaction of the vanadium species with PCHs support [20]. Nevertheless, the reduction temperature of vanadium catalysts supported on PCHs is lower than that of bulk V_2_O_5_, which indicates that the particle size and the interaction between active site and support are key factors in the reducibility of vanadium species.

Considering that the reduction of V^5+^ to V^0^-species requires an H_2_ consumption of 13,745 μmol g^−1^, the H_2_ consumption for the reduction of the V_2_O_5_-bulk well below the theoretical values since the H_2_ uptake is only 5659 μmol g^−1^. This fact supposes a partial reduction of the V^5+^-species, as previously indicated, since it is only possible reduce the V^5+^ to V^3+^-species in this temperature range [10,20]. Taking into account this partial reduction (V^5+^→V^3+^), the H_2_ uptake is very close to the theoretical value (5498 μmol g^−1^). In the case of the *x*-PCH-*y*V catalysts, the partial reduction of V-species (V^5+^→V^3+^) is also observed, as indicates the Supplementary Materials Table S1. The obtained data also reveals that H_2_ consumption of the catalysts is very close to the theoretical values for the partial reduction of the V^5+^-species to V^3+^-species.

## 4. Catalytic Results

**Figure 5:**
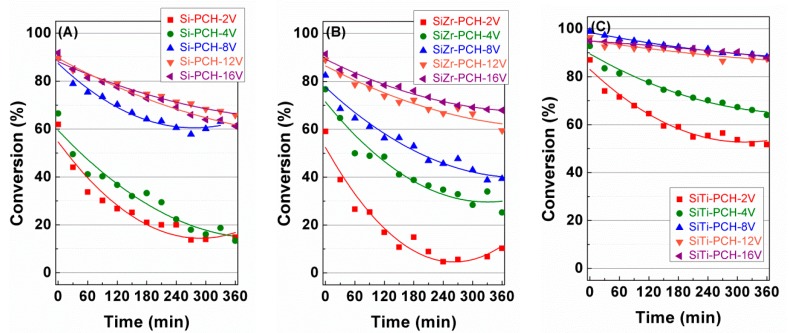
Variation of the H_2_S conversion with the time on stream (TOS) obtained for V-based catalysts supported on Si-PCH (**A**), SiZr-PCH (**B**) and SiTi-PCH (**C**). Reaction conditions: 0.05 g of catalyst; total flow of 130 mL min^−1^; reaction temperature of 180 °C; H_2_S/air/He molar ratio of 1.2/5.0/93.8.

The *x*-PCH-*y*V catalysts were evaluated for 360 min at 180 °C with a reactant molar composition of H_2_S/air/He = 1.2/5.0/93.8 in the partial oxidation of hydrogen sulfide to sulfur (Figure 5). All catalysts display the highest H_2_S conversion values in the initial stage of the reaction although the conversion decreases with the time on stream mainly for the catalysts with the lowest vanadium content. The catalytic results suggest that the use of SiTi-PCH as support minimizes the deactivation of the catalyst with time on stream, reaching a conversion value of 89% for SiTi-PCH-16V catalyst after 360 min on stream, while Si-PCH as support displays a highest conversion of 66% for Si-PCH-12V catalyst and SiZr-PCH exhibits a highest conversion value of 63% for SiZr-PCH-16V catalyst after 360 min on stream.

**Figure 6:**
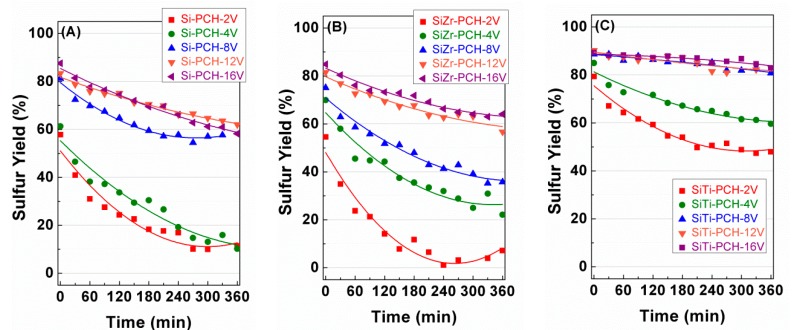
Variation of the sulfur yield with the time on stream (TOS) obtained for V-based catalysts supported on Si-PCH (**A**), SiZr-PCH (**B**) and SiTi-PCH (**C**). Reaction conditions: 0.05 g of catalyst; total flow of 130 mL min^−1^; reaction temperature of 180 °C; H_2_S/air/He molar ratio of 1.2/5.0/93.8.

**Figure 7:**
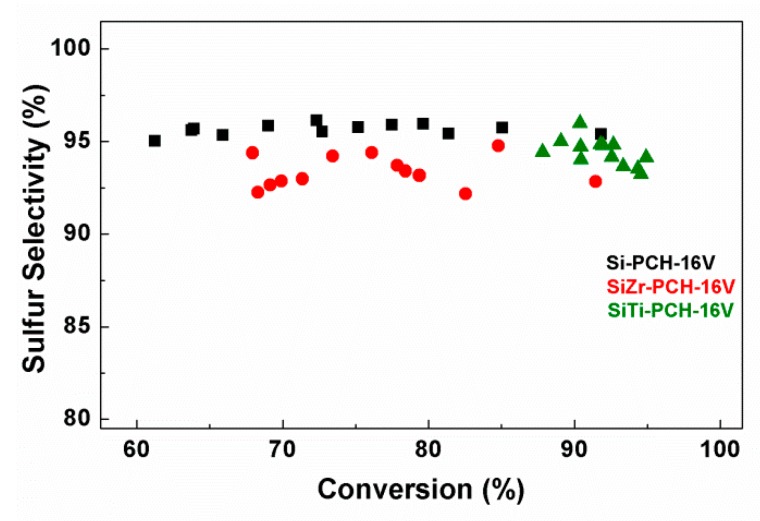
Variation of the selectivity to sulfur with the H_2_S conversion obtained over Si-PCH16, SiZr-PCH-16V and SiTi-PCH-16V catalysts.

With regard to the yield (Figure 6), all catalysts show sulfur elemental yield similar to those shown in the H_2_S conversion. This fact indicates that sulfur elemental is the main product in all cases and the undesired SO_2_ hardly is generated due to the use of a relatively low temperature for these reactions (180 °C), avoiding the total oxidation of H_2_S to SO_2_ [8,10]. Figure 7 plots the influence of the sulfur selectivity with the H_2_S conversion for *x*-PCH-16V catalysts, as representative catalysts. As was mentioned previously, elemental sulfur is the main product maintaining a similar selectivity close to between 92.5–95% when the varying the H_2_S conversion and displaying SO_2_ selectivities of ca. 5–7.5% in all cases.

## 5. Evolution of the Active Phase

In order to gain further insight into the possible active phase modification, XRD, Raman spectroscopy and XPS were carried out for catalysts after the catalytic tests.

**Figure 8:**
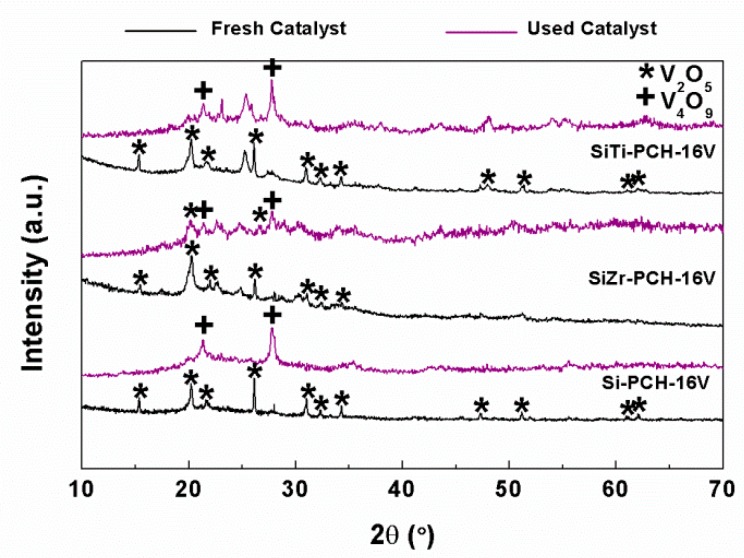
Comparison of the X-ray diffractograms of the Si-PCH16, SiZr-PCH-16V and SiTi-PCH-16V catalysts before and after selective oxidation of H_2_S reaction. Reaction conditions: 0.05 g of catalyst; total flow of 130 mL min^−1^; reaction temperature of 180 °C; H_2_S/air/He molar ratio of 1.2/5.0/93.8.

XRD patterns of the spent catalysts (Figure 8) show how the diffraction peaks attributed to the V_2_O_5_ species disappears, arising new diffraction peaks, located at 21.3 and 27.7° (PDF N: 01-071-2248), which have been assigned to the presence of V_4_O_9_ species due to the partial reduction of V_2_O_5_ species during the catalytic tests [8]. It has been reported in the literature that this phase is relatively stable, although in time it evolves to V_2_O_5_ again when in contact with the air [9]. Therefore, it has been proposed that V_4_O_9_ species is the active species in the selective oxidation of H_2_S due to V_4_O_9_ crystals display high capacity to transport lattice oxygen improving the redox behavior of these catalytic systems [8,10].

The diffractograms also reveal that V_2_O_5_ reflections disappear completely to generate V_4_O_9_ species in the case of Si-PCH-16V and SiTi-PCH-16V catalysts. However, in the case of the SiZr-PCH-16V the reduction from V_2_O_5_ to V_4_O_9_ is only partial. In this sense, some authors have pointed out that the formation of reduced vanadium species such as VO_2_, V_2_O_3_, V_3_O_7_ and V_4_O_9_ become stable in the selective H_2_S oxidation into elemental sulfur [37]. In addition, these authors have pointed out that the V_2_O_5_-species with high crystallinity tend to be highly selective towards the undesired SO_2_, while the partially reduced vanadium species, such as V_4_O_9_, promote partial oxidation to form elemental sulfur [37].

Previous researches have established that the presence of acid sites favors the partial oxidation of H_2_S [4,21]. Basing on this premise, SiTi-PCH-16V and SiZr-PCH-16V catalysts should display higher conversion values than Si-PCH-16V catalyst, due to, as was mentioned previously, the incorporation of heteroatoms in the silica pillars generates acid sites. The catalytic results reveal a high conversion values for the SiTi-PCH-16V catalyst, however the SiZr-PCH-16V displays lower conversion values than expected. This fact can be attributed to the fact that the SiZr-PCH-16V catalyst displays a mixture of V_2_O_5_ and V_4_O_9_ species while the SiTi-PCH-16V catalyst only displays V_4_O_9_ species, which are the active species in the selective oxidation of H_2_S. The presence of lower amount of V_4_O_9_ species in the SiZr-PCH-16V catalyst can be related to the coexistence of V_2_O_5_ species and ZrV_2_O_7_, which are not so easily reducible, leading to lower activity and higher deactivation. 

Finally, in the case of SiTi-PCH-*y*V and SiZr-PCH-*y*V catalysts before the reaction, other phases due to the segregation of TiO_2_ in the case of the SiTi-PCH-*y*V catalysts or the formation of ZrV_2_O_7_ species by the extraction of ZrO_2_ of the pillars and subsequent interaction with the vanadium species in the case of the SiZr-PCH-yV catalysts were detected. These reflections remain unaltered after the catalytic test.

**Figure 9:**
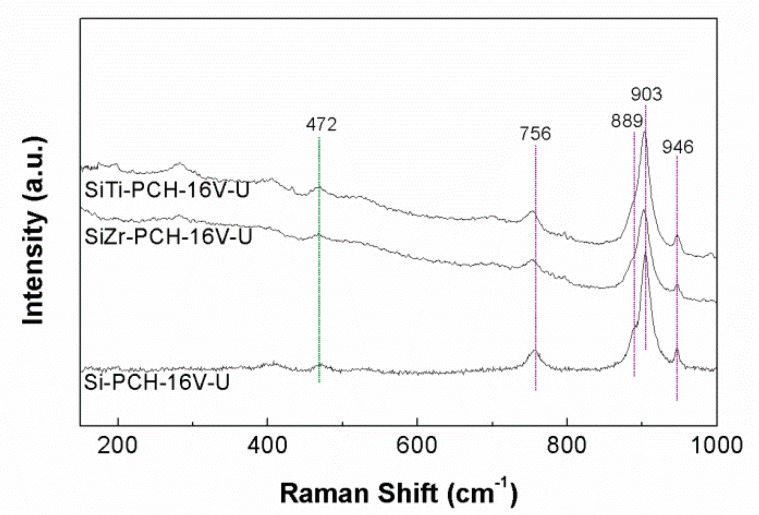
Raman spectra of Si-PCH-16V, SiZr-PCH-16V and SiTi-PCH16V catalysts selective oxidation of H_2_S reaction. Reaction conditions: 0.05 g of catalyst; total flow of 130 mL min^−1^; reaction temperature of 180 °C; H_2_S/air/He molar ratio of 1.2/5.0/93.8.

The Raman spectra of the *x*-PCH-16V spent catalysts (Figure 9) also reveal the loss of the typical bands of V_2_O_5_ and the presence of new bands located about 756, 889, 903 and 946 cm^−1^ attributed to the presence of V_4_O_9_ species [8,38], confirming to that observed in the XRD data for the spent catalysts. In this sense, a previous research has also established the presence of V_4_O_9_ by in operando X-ray absorption spectroscopy (XAS) during the reaction in vanadium oxide catalysts supported in mesoporous zirconium phosphate [9]. The absence of a band, about 614 cm^−1^, discounts the presence of VO_2_ species which could be formed by the prolonged reduction of V_2_O_5_ species, although the low reaction temperature and the low reductive character of the reaction conditions can favor the formation of V_4_O_9_ species [39]. However, these V_4_O_9_ are not stable, since they tend to oxidize to V_2_O_5_ in contact with air over time.

In addition, the band located about 472 cm^−1^ is related to the presence of S-S polymeric sulfur species [40], although it has not been observed in bands attributed to sulfur vanadate from XRD data and Raman spectra in any catalyst [8,41].

**Figure 10:**
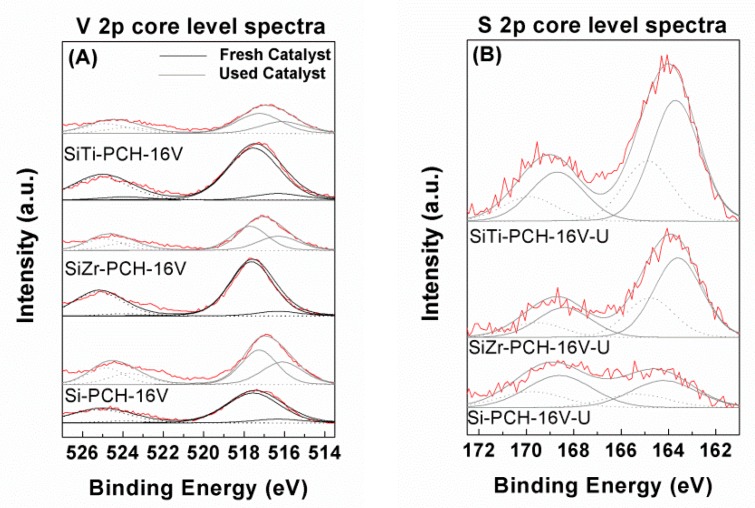
V 2*p* core level spectra of Si-PCH-16V, SiZr-PCH-16V and SiTi-PCH16V catalysts before and after selective oxidation of H_2_S reaction (**A**) and S 2p core level spectra of Si-PCH-16V, SiZr-PCH-16V and SiTi-PCH16V catalysts after selective oxidation of H_2_S reaction (**B**). Reaction conditions: 0.05 g of catalyst; total flow of 130 mL min^−1^; reaction temperature of 180 °C; H_2_S/air/He molar ratio of 1.2/5.0/93.8.

The XPS spectra of the used catalysts reveal an increasing of the relative intensity of the band located between 516.3–516.6 eV in the V 2*p*_3/2_ region (Figure 10A) attributed to the presence of V^4+^ species, which confirms the partial reduction of the vanadium species during the catalytic process [10]. In the S 2 *p*_3/2_ region (Figure 10B), new bands arise after the catalytic test, located about 163.6–163.9 eV attributed to elemental sulfur and between 168.4–168.7 eV assigned to sulfate species [10]. From the spectra parameters (Table 2), it can be observed how SiZr-PCH-16V and SiTi-PCH-16V catalysts show a higher contribution to elemental sulfur than Si-PCH-16V catalyst. In addition, SiZr-PCH-16V and SiTi-PCH-16V catalysts display higher sulfur content on the surface than Si-PCH-16V. This fact can be attributed to the acidity of the catalysts due to the presence of acid sites that favor the selective oxidation of H_2_S, favoring the deposition of sulfur on the surface and leading to the deactivation of the catalysts [4]. Finally, the V/(Si + Al + Zr + Ti) molar ratios reveal that Si-PCH-16V suffers a sintering, while both SiZr-PCH-16V and SiTi-PCH-16V catalysts hardly suffer sintering, so the decrease of the conversion in the Si-PCH-16V catalyst can also be attributed to the loss of available vanadium sites.

## 6. Conclusions

Several porous clay heterostructures (PCHs), formed by silica, silica-zirconia or silica-titania pillars, have been used as catalytic supports to disperse V_2_O_5_ species, which have shown to be active in the selective oxidation of H_2_S to elemental sulfur.

The catalytic behavior of these catalysts shows how the catalytic activity and the stability improves for larger V content, attaining the highest yield towards sulfur elemental when a 16 wt.% of V was incorporated in the PCHs. The catalysts supported on PCHs with silica-titania in the pillars are more active and more resistant to deactivation processes by the deposition of sulfur and sulfates on the catalyst surface, reaching a highest H_2_S conversion of 89% and total selectivity to elemental sulfur for the SiTi-PCH-16V after 360 min at 180 °C. Nonetheless, other catalysts supported on SiTi-PCH and lower vanadium content have also shown excellent activity and resistance to deactivation processes.

The characterization of the spent catalysts has shown the formation of a new crystallographic phase (V_4_O_9_), which seems to be involved in the selective oxidation of H_2_S to elemental sulfur due to the redox properties of this phase under the reaction conditions.

## Figures and Tables

**Table 1:** Textural and acid parameters of the starting montmorillonite, support and catalysts.

Sample	S_BET_ (m^2^ g^−1^)	*t*-Plot_microp._ (m^2^ g^−1^)	V_P_ (cm^3^ g^−1^)	V_Pmicrop._ (cm^3^ g^−1^)	D_p_ (nm)	µmol NH_3_ g^−1^	µmol NH_3_ m^−2^
Montmorillonite	49	18	0.109	0.009	12.4	125	2.55
Si-PCH	643	460	0.773	0.280	5.5	308	0.48
Si-PCH-2V	555	367	0.773	0.219	5.6	517	0.93
Si-PCH-4V	483	316	0.751	0.191	7.0	671	1.39
Si-PCH-8V	374	224	0.612	0.136	7.6	711	1.90
Si-PCH-12V	241	83	0.549	0.047	10.5	587	2.44
Si-PCH-16V	147	43	0.400	0.021	12.8	417	2.84
PCH-SiZr	608	382	0.829	0.212	6.8	460	0.76
SiZr-PCH-2V	477	290	0.813	0.157	8.8	614	1.34
SiZr-PCH-4V	420	253	0.675	0.138	8.3	874	2.08
SiZr-PCH-8V	329	179	0.571	0.097	8.9	810	2.46
SiZr-PCH-12V	216	86	0.543	0.047	11.7	657	3.04
SiZr-PCH-16V	161	63	0.442	0.034	12.4	547	3.39
SiTi-PCH	562	287	0.796	0.164	6.8	395	0.70
SiTi-PCH-2V	472	230	0.836	0.130	8.8	559	1.18
SiTi-PCH-4V	405	170	0.750	0.097	8.5	599	1.48
SiTi-PCH-8V	262	54	0.607	0.033	9.2	799	3.04
SiTi-PCH-12V	158	17	0.544	0.008	15.1	677	4.28
SiTi-PCH-16V	109	11	0.440	0.004	17.5	603	5.53

**Table 2:** Atomic concentrations (estimated by XPS) of the V-based catalysts.

Sample	Atomic Concentrations						
	O 1*s*	Si 2*p*	Al 2*p*	Zr 3*d*	Ti 2*p*	V 2*p*	S 2*p*
Si-PCH-2V	66.30	30.85	2.19	-	-	0.67	-
Si-PCH-4V	66.39	30.75	1.95	-	-	0.90	-
Si-PCH-8V	67.14	30.04	1.68	-	-	1.15	-
Si-PCH-12V	65.78	31.11	1.35	-	-	1.76	-
Si-PCH-16V	65.53	30.96	1.70	-	-	1.81	-
Si-PCH-16V-u	64.93	28.89	2.26	-	-	3.25	0.67
SiZr-PCH-2V	67.03	28.18	2.51	1.81	-	0.40	-
SiZr-PCH-4V	66.86	27.47	2.73	1.90	-	1.04	-
SiZr-PCH-8V	66.35	26.86	3.13	1.83	-	1.89	-
SiZr-PCH-12V	66.55	26.49	3.16	1.54	-	2.26	-
SiZr-PCH-16V	65.71	27.45	2.93	1.23	-	2.76	-
SiZr-PCH-16V-u	66.59	25.14	2.67	1.26	-	2.83	1.51
SiTi-PCH-2V	66.59	28.88	3.10	-	0.98	0.53	
SiTi-PCH-4V	67.67	28.44	3.12	-	0.81	0.56	
SiTi-PCH-8V	67.31	27.49	3.09	-	0.71	1.40	
SiTi-PCH-12V	66.30	27.76	3.07	-	0.48	2.38	
SiTi-PCH-16V	65.97	27.94	2.63	-	0.45	3.02	
SiTi-PCH-16V-u	65.35	26.61	2.69	-	0.51	3.12	1.72

**Table 3:** Superficial atomic ratios and V 2*p*_3/2_ and S 2*p*_3/2_ binding energies for the V-based catalysts.

Sample	Superficial Atomic Ratio		Binding Energy (eV)			
	V/(Si + Al + Zr + Ti)	V/S	V^5+^	V^4+^	SO_4_^2−^	S^0^
Si-PCH-2V	0.020	-	517.5 (78.3%)	516.3 (21.7%)	-	-
Si-PCH-4V	0.028	-	517.7 (80.5%)	516.5 (19.5%)	-	-
Si-PCH-8V	0.036	-	517.6 (82.4%)	516.1 (17.6%)	-	-
Si-PCH-12V	0.054	-	517.1 (86.2%)	516.0 (13.8%)	-	-
Si-PCH-16V	0.055	-	517.1 (87.2%)	516.1 (12.8%)	-	-
Si-PCH-16V-u	0.104	4.850	517.4 (60.4%)	516.3 (39.6%)	168.7 (46.5%)	163.9 (54.5%)
SiZr-PCH-2V	0.012	-	517.5 (84.8%)	516.2 (15.2%)	-	-
SiZr-PCH-4V	0.032	-	517.3 (83.1%)	516.1 (16.9%)	-	-
SiZr-PCH-8V	0.059	-	517.7 (86.6%)	516.3 (13.4%)	-	-
SiZr-PCH-12V	0.072	-	517.6 (91.9%)	516.1 (8.1%)	-	-
SiZr-PCH-16V	0.087	-	517.6 (92.0%)	516.3 (8.0%)	-	-
SiZr-PCH-16V-u	0.097	1.874	517.7 (62.8%)	516.5 (37.2%)	168.4 (27.4%)	163.6 (72.6%)
SiTi-PCH-2V	0.016	-	517.3 (82.6%)	516.0 (17.4%)	-	-
SiTi-PCH-4V	0.017	-	517.4 (88.4%)	516.1 (11.6%)	-	-
SiTi-PCH-8V	0.044	-	517.5 (88.0%)	516.3 (12.0%)	-	-
SiTi-PCH-12V	0.076	-	517.6 (88.8%)	516.3 (11.2%)	-	-
SiTi-PCH-16V	0.097	-	517.5 (88.8%)	516.2 (11.2%)	-	-
SiTi-PCH-16V-u	0.105	1.814	517.7 (62.8%)	516.6 (37.2%)	168.6 (29.0%)	163.7 (71.0%)

## Supplementary Materials

The following are available online at http://www.mdpi.com/1996-1944/11/9/1562/s1, Table S1. H2-consumption, estimated from the H_2_-TPR profiles, of the *x*-PCH-*y*V catalysts. Figure S1. X-ray diffractogram of the starting bentonite. Figure S2. High angle diffractogram of the Si-PCH, SiZr-PCH and SiTi-PCH. Figure S3. N2 adsorption-desorption isotherms of the supports (A), of the V-based catalysts supported on Si-PCH (B), of the V-based catalysts supported on SiZr-PCH (C) and of the V-based catalysts supported on SiTi-PCH (D). Figure S4. Pore-size distribution (estimated by the DFT method) of the supports (A), of the V-based catalysts supported on Si-PCH (B), of the V-based catalysts supported on SiZr-PCH (C) and of the V-based catalysts supported on SiTi-PCH (D).
