# Supplementary material for: Selective Oxidation of Hydrogen Sulfide to Sulfur Using Vanadium Oxide Supported on Porous Clay Heterostructures (PCHs) Formed by Pillars Silica, Silica-Zirconia or Silica-Titania
Source: Materials (Basel). 2018 Aug 30;11(9):1562. doi: 10.3390/ma11091562 (PMC6163609; doi:10.3390/ma11091562)
Supplement: Supplementary file 1 [file materials-11-01562-s001.zip › materials-345512-SI.docx]

Selective Oxidation of Hydrogen Sulfide to Sulfur Using Vanadium Oxide Supported on Porous Clay Heterostructures (PCHs) Formed by Pillars Silica, Silica-Zirconia or Silica-Titania

Juan Antonio Cecilia *, M. Dolores Soriano, Alejandro Natoli, Enrique Rodríguez-Castellón and José Manuel López Nieto

**Table S1.** H_2_-consumption, estimated from the H_2_-TPR profiles, of the *x*-PCH-*y*V catalysts.

| Vanadium Content (wt.%) | Support | | | H_2_ consumption (μmol g^−1^)  (theoretical values)^*^ |
| --- | --- | --- | --- | --- |
|  | **Si-PCH**  **(μmol g^−1^)** | **SiZr-PCH**  **(μmol g^−1^)** | **SiTi-PCH**  **(μmol g^−1^)** |  |
| 2 wt.% | 188 | 199 | 180 | 196 |
| 4 wt.% | 398 | 411 | 407 | 409 |
| 8 wt.% | 840 | 860 | 842 | 853 |
| 12 wt.% | 1320 | 1348 | 1323 | 1338 |
| 16 wt.% | 1855 | 1887 | 1860 | 1869 |

^*^ Theoretical value considering the reduction V^5+^→V^3+^.





**Figure S1.** X-ray diffractogram of the starting bentonite.





**Figure S2.** High-angle diffractogram of the Si-PCH, SiZr-PCH and SiTi-PCH.

|  |  |
| --- | --- |
|  |  |

**Figure S3.** N_2_ adsorption-desorption isotherms of the supports (**A**), of the V-based catalysts supported on Si-PCH (**B**), of the V-based catalysts supported on SiZr-PCH (**C**) and of the V-based catalysts supported on SiTi-PCH (**D**).

|  |  |
| --- | --- |
|  |  |

**Figure S4.** Pore-size distribution (estimated by the DFT method) of the supports (**A**), of the V-based catalysts supported on Si-PCH (**B**), of the V-based catalysts supported on SiZr-PCH (**C**) and of the V-based catalysts supported on SiTi-PCH (**D**).
